# Spin-Dependent First-Principles Study on Optoelectronic Properties of Neodymium Zirconates Pyrochlores Nd_2_Zr_2_O_7_ in Fd-3m and Pmma Phases

**DOI:** 10.3390/molecules27175711

**Published:** 2022-09-05

**Authors:** Azhar Qayyum, Sikander Azam, Ali H. Reshak, Jehan Akbar, Zeesham Abbas, Haseen Ullah, Muhammad M. Ramli

**Affiliations:** 1 Department of Physics, Hazara University, Mansehra 21300, Pakistan; 2 Faculty of Engineering and Applied Sciences, Department of Physics, RIPHAH International University, I-14 Campus, Islamabad 42000, Pakistan; 3 Physics Department, College of Science, University of Basrah, Basrah 61004, Iraq; 4 Department of Instrumentation and Control Engineering, Faculty of Mechanical Engineering, CTU in Prague, Technicka 4, 616607 Prague, Czech Republic; 5 Center of Excellence Geopolymer and Green Technology, (CEGeoGTech), University Malaysia Perlis, Kangar 01007, Malaysia; 6 Glasgow College, University of Electronic Science and Technology of China, Chengdu 610000, China; 7 Department of Nanotechnology and Advanced Materials Engineering, Sejong University, Seoul 05006, Korea; 8 Department of Basic Sciences and Islamiat, University of Engineering & Technology, Peshawar 25000, Pakistan

**Keywords:** zirconates pyrochlores, density functional theory, GGAU approximation, electronic band structure, optical properties

## Abstract

Rare-earth zirconate pyrochlores (RE_2_Zr_2_O_7_) are of much fundamental and technological interest as optoelectronic, scintillator and thermal barrier coating materials. For the first time, we report the detailed optoelectronic properties of rare-earth zirconates Nd_2_Zr_2_O_7_ in both, i.e., for spin up and spin down states, via the use of first-principles density functional theory (DFT) procedure. To obtain the desired optoelectronic properties, we used a highly accurate method called full-potential linearized augmented plane wave (FPLAPW) within the generalized gradient approximation (GGA), parametrized with Hubbard potential *U* as an exchange-correlation function. The band gaps predicted for Nd_2_Zr_2_O_7_ were of the order 2.4 eV and 2.5 eV in Fd-3m and Pmma symmetrical phases, respectively. For both the phases, our research involved a complete examination of the optical properties of Nd_2_Zr_2_O_7_, including extinction coefficient, absorption coefficient, energy loss, function, reflectivity, refractive index, and real optical conductivity, analyzed in the spectral range from 0.0 eV to 14 eV. The calculated optical properties in both phases showed a considerable spin-dependent effect. The electronic bonding characteristics of different species in Nd_2_Zr_2_O_7_ within the two crystal symmetries were explored via the density distribution mapping of charge.

## 1. Introduction

Due to their unique physical and chemical properties, such as storage of nuclear waste, electrolytes in solid oxide fuel cells, oxygen sensors, etc., pyrochlore oxides (A_2_B_2_O_7_) have risen to the forefront of intense research [1,2,3]. The disordering of the anions and cations in the pyrochlore lattice determines the structure and characteristics of pyrochlores. The Fd-3m space group is where the ordered pyrochlore resides. The simple fluorite structure is modified in an orderly manner to form the pyrochlore structure [1,4]. Pyrochlores have been known to undergo a number of phase transitions, including the crystalline to amorphous transition, phase separation, and an order–disorder structural alteration when exposed to high temperatures or pressure [1,5,6,7,8,9,10]. In particular the phenomenon of order–disorder phase transition from pyrochlore to defect-fluorite structure is caused by the existence of oxygen vacancies. Due of the significant changes in characteristics as a result of the disordering of the A and B site cations and oxygen vacancies, the phase transition investigations of pyrochlores under extreme circumstances are of particular interest. It is understood that altering the composition, raising the temperature, or exposing the pyrochlores to high-energy irradiations can induce the order–disorder transition in them [4]. Another intriguing but underdeveloped research field is the examination of structural modifications in the pyrochlore lattice at high pressure. High pressure causes phase changes by strengthening intermolecular interactions between molecules, changing intermolecular bonding, and enhancing the disordering of oxygen vacancies.

The Nd_2_Zr_2_O_7_ (NZO) has been extensively considered for nuclear waste host material also [5,6,7]. It gives the benefits of chemical stability, thermodynamic stability, excellent resistance to the radiations, and much more capablity to contain a higher quantity of actinides in the lattices [8,9,10]. By considering the valence, ionic radius, and additional number of electronic orbits, Nd^3+^ ion (1.1 Å) can be considered as an option for actinides Pu^3+^ (1.0 Å), U^3+^ (1.06 Å), Am^3+^ (1.01 Å), and Th^3+^ (0.9 Å). By completely changing the Gd^3+^ ion in Gd_2_Zr_2_O_7_, the replicated NZO solidifies to form a highly radioactive waste, making it a very interesting field of research [11,12,13], and in particular the effects under the influence of heavy ion irradiation has been widely investigated. Sickafus predicted that the radiation acceptance of NZO is dependent upon the cation radius based on examinations of similar complex oxides under the irradiations of heavy ions (350 KeV X e++) [14]. After that, the radiation acceptance response of NZO has been significantly improved through the continual efforts of scientists around the world.

Patel et al. found that NZO may be amorphous under the irradiations of high-energy heavy ions (i.e., Au at 120 MeV, I at 90 MeV, Ni at 70 MeV, and U at 119 MeV) [15,16,17,18]. 

Recently, pyrochlore materials have garnered significant research interest due to their peculiar properties, such as structural flexibility [19,20], high dielectric constant [21], high radiation stability [22], order–disorder phase transition [23], etc. A number of these properties make the material a promising candidate for self-activated phosphors, luminescence hosts, scintillators, nuclear waste hosts, fuel cells for solid oxide, catalysis, actinide speciation, and magnetism, etc. [19,24,25,26,27,28,29,30,31,32,33,34,35]. Amongst various pyrochlores, the compound NZO has been the subject of much consideration due to its promise for diverse applications, including in solid-state lasers [16], photocatalysis [36], for small actinide transmutation as a host [37], coatings [38], magnets [39], etc. The theoretical calculation was carried out for the first time on an NZO compound by Xiao et al. [40] and he predicted the transition of phase at high pressure. To prepare the NZO powder, numerous techniques/methods have been applied, for example the solid-state technique [41], the co-precipitation method [39], sol-gel method [42], aqueous chemical synthesis technique [43], etc. The details of preparation methods for A_2_B_2_O_7_ compounds can be found in [44]. The stabilization of the NZO compound in the fluorite phase was observed at 900 °C but the phase transition to ideal pyrochlore occurred at 1000–1400 °C [43]. There is controversy regarding the structure of NZO [42,45,46,47]. Lee et al. [34], and Bhattacharya et al. [35] synthesized NZO at 750 and 700 °C, respectively, and reported that it is stable in the fluorite phase. However, on the other hand, Zhang and his collaborators [46] reported that at 600 °C, the NZO compound weakly crystallized in the pyrochlore phase. However, according to Rao et al. [47], the structure of NZO is not certain at 500 °C. This structural uncertainty is ascribed to the fact that the crystallinity of the samples was very small in terms of the size of the nanoparticles, which required a wide XRD pattern. 

As is obvious from the literature presented above, most of the previous studies on NZO have been done experimentally. Only a few works on NZO have been published using first-principles methods, mainly reporting on structural and energy band properties [48,49,50]. In particular, as per our knowledge, any systematic study has rarely been done on the optical properties of the compound. This state of affairs motivated us to investigate the detailed optoelectronic properties of the NZO compound using the first-principles density functional theory (DFT) procedure outlined below.

## 2. Materials and Methods

### Computational Methodology

In this study, our DFT calculations have been derived from full-potential linearized augmented plane wave (FPLAPW) methodology, within the generalized gradient approximation (GGA), parametrized with Hubbard potential *U* as an exchange-correlation functional while implemented in WIEN-2k code [51]. The adopted GGA+U approach with Hubbard’s potential U valued at 7.0 eV demonstrates accurate results for the band-gap energy calculation as compared to the conventional DFT approach. The muffin-tin radii RMT for Nd_2_Zr_2_O_7_ compound was put as 2.0 Bohr for the neodymium, zirconium, and oxygen atoms. 

Moreover, we have taken the Gaussian factor and angular momentum (Gmax = 12, ι = 10). The convergence of energy/charge has been done up to the 10^−5^ Ry by the iteration process. The default value of kinetic energy cut off (−6.0 Ry) was taken during all the calculations. We adopted the Kramers–Kronig formalism to compute optical properties.

The plane wave cutoff was set so that RMT × K_MAX_ = 7, where forces converge on the atoms, was validated. For the structural, electronic, and optical properties of compound Nd_2_Zr_2_O_7_, a 16 × 16 × 15 k-point mesh and GGA+U functional was used to relax the compound structure.

The linear optical properties of Nd_2_Zr_2_O_7_ were studied by the program “OPTIC” [52], integrated in WIEN-2k code. The complex dielectric function *ε*(*ω*) is derived from the Kramers–Kronig relation [53]. The optical conductivity and the energy loss function can be directly calculated from ε_1_(ω) and ε_2_(ω) [54]. The electron energy loss spectra of compound Nd_2_Zr_2_O_7_ at different edges were estimated with the X-ray absorption module of the WIEN-2k code. 

To describe the linear optical susceptibility of the crystal, we needed dielectric components and following expression was used:

(1) $$\varepsilon_{2}{}^{ij}\left(\omega \right)=\frac{4\pi^{2}e^{2}}{Vm^{2}\omega^{2}}\times \underset{knn^{\prime }\sigma }{\sum }\langle kn\sigma |p_{i}|kn^{\prime }\sigma \rangle \langle kn^{\prime }\sigma |p_{j}|kn\sigma \rangle \times f_{kn}\left(1-f_{kn^{\prime }}\right)\sigma \left(E_{kn^{\prime }}-{Ε}_{kn}-\hbar \omega \right)$$

where *e* stands for the charge of the electron and *m* is its mass, the angular frequency is (ω), the unit cell volume is represented by V, momentum operator is represented by p, and the crystals wave function is noted as *knσ*. The transitions from occupied VB states to unoccupied states are denoted by *f*_kn_. From the total density of states, the term δ ($E_{kn^{\prime }}$ − $E_{kn}$− ħω) gives optimizing parameters to evaluate total energy. In order to understand more about the corresponding transitions and to compare the optical transition dipole matrix, we extracted the real part of dielectric function from its imaginary part by using the Kramers–Kronig relation:

(2) $$\varepsilon_{1}\left(\omega \right)=1+\frac{2}{\pi }Ρ\int_{0}^{\infty }\frac{\omega^{\prime }\varepsilon_{2}\left(\omega^{\prime }\right)}{\omega^{’2}-\omega^{2}}d\omega^{\prime }$$

where P stands for the principal value of the integral.

We have also investigated the other optical properties from ε_1_(ω) and ε_2_(ω)_,_ including absorption coefficient *I^ave^*(ω), optical reflectivity coefficient *R^ave^*(ω), and electron energy loss *L^ave^*(ω).

(3) $$\alpha^{ij}\left(\omega \right)=\frac{2\omega k^{ij}\left(\omega \right)}{c}$$

(4) $$R^{ij}\left(\omega \right)=\frac{{\left(n^{ij}-1\right)}^{2}+k^{ij}}{{\left(n^{ij}+1\right)}^{2}+k^{ij}}={\left|\frac{\sqrt{\varepsilon_{1}^{ij}+i\varepsilon_{1}^{ij}}}{\sqrt{\varepsilon_{1}^{ij}+i\varepsilon_{1}^{ij}}}\frac{-1}{+1}\right|}^{2}$$

(5) $$L^{ij}\left(\omega \right)=-Im{\left(\varepsilon^{-1}\right)}^{ij}=\frac{\varepsilon_{1}^{ij}\left(\omega \right)}{\varepsilon_{1}^{ij}{\left(\omega \right)}^{2}+\varepsilon_{2}^{ij}{\left(\omega \right)}^{2}}$$

## 3. Results and Discussion

### 3.1. Crystallographic Structure

**Figure 1:**
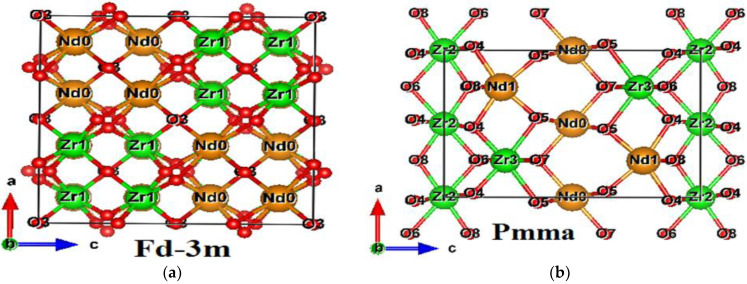
Crystal structures for (**a**) Fd-3m and (**b**) Pmma phases of Nd_2_Zr_2_O_7_ compound.

The two different space group crystal structures of the Nd_2_Zr_2_O_7_ compound are shown in Figure 1. The calculated (optimized) lattice parameters and fractional coordinates for Nd_2_Zr_2_O_7_ compound in Fd-3m and Pmma are given in Table 1 and Table 2 [53,55]. Hence, the calculated structural parameters were almost equal to the experimental values. Previously, calculated lattice constant for compound Nd_2_Zr_2_O_7_ in crystal symmetry was found to be 10.68 [50] based on the LDA+U method, and 10.74 Å [53] using the GGA+U method. The stability of the materials has been found by calculating the compound binding energy using the following equation:

(6) $$E_{BE}=E_{TOT}\left(Nd_{2}{\mathrm{Zr}}_{2}O_{7}\right)-E_{1}\left(2\left(Nd\right)\right)-E_{2}\left(2\left(Zr\right)\right)-E_{3}\left(7\left(O\right)\right)$$

where is *E_TOT_*, *E*_1_, *E*_2_, and *E*_3_ are the total energy of the Nd_2_Zr_2_O_7_ system, for the Nd atom, for the Zr atom, and for the O atom. The binding energy formation values for Fd-3m and Pmma were −3.860 and −3.074. If a compound has negative binding energy, it is considered as thermodynamically stable. It is evident that our investigated materials were thermodynamically stable as they had negative values of formation energy. Fd-3m had the lowest value, indicating a more thermodynamically stable material.

### 3.2. Electronic Band Structure

**Figure 2:**
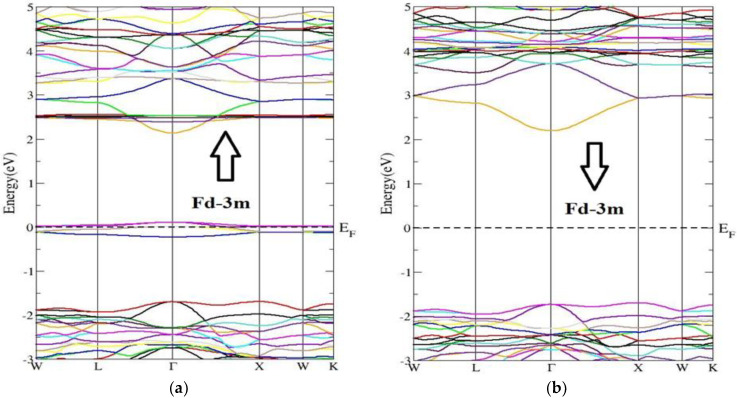
Energy band structure of Nd_2_Zr_2_O_7_ compound in Fd-3m phase for (**a**) the spin-up direction (↑) and (**b**) spin-down (↓) direction.

**Figure 3:**
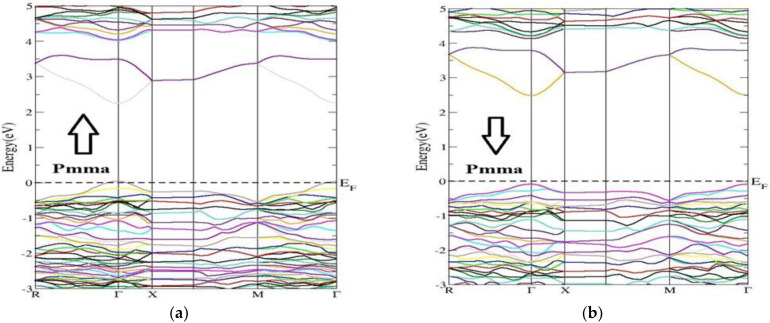
Energy band structure of the Nd_2_Zr_2_O_7_ compound in the Pmma phase for the (**a**) the spin-up direction (↑) and (**b**) spin-down (↓) direction.

Most of the physical properties of solids are associated with the electronic band structure, so the study of the electronic band structure is most important. The electronic energy band-gap properties and their values mainly decide the charge transport and optical features of semiconductors. The electronic band structure is calculated in a highly symmetrical direction along the first Brillion zone (BZ) of the lattice. The highly symmetrical points are of great importance, correlated to the Brillion Zone (BZ) of the primitive reciprocal lattice unit cell. The coordinates depend upon the symmetrical groups to which the crystal structure belongs. The unit-primitiv cells and their coefficients are related to the unit vectors defined by the lattice parameters, for both real and reciprocal lattices. The main investigated features, energy band structure and electronic density of the states (partial and total) of the compound Nd_2_Zr_2_O_7_ for both the phases and for both the spins (up and down), are shown in Figure 2 and Figure 3, respectively. The main difference was the band-gap energy. The minima of the conduction band (CBM) and the maxima of valance band (VBM) were located at the single point Ӷ of the Brillion zone (BZ) for the spin up and spin down in both the space groups, ensuring direct band-gap materials. The materials with direct band gaps are called active semiconductors. The response of the indirect band-gap materials to the optical excitation was very weak, particularly at the absorption threshold. The reason is that the CBM and the VBM take different locations of the BZ for indirect band-gap materials. 

The investigated band gaps of the NZO compound for both the space group Fd-3m and Pmma were 2.09 eV and 2.39 eV, respectively, for spin up and were equal to 3.90 eV and 2.40 eV, respectively, for spin down. The band gap for spin down was very broad as compared to that of spin up. On the other hand, the band gap for Pmma spin up and down were almost equal. In the case of the spin up state with symmetry, a small intermediate band lay around the Fermi level. Such materials with intermediate bands find applications in the intermediate band solar cells [53]. For comparison, previously reported band gaps for Nd_2_Zr_2_O_7_ have been of order 3.72 (form first-principles molecular dynamic calculations) [48], 2.67 (from first-principles DFT procedure) [49], and 4.0 (using LDA+U method) [50]. 

### 3.3. Electronic Density of States

The electronic density of state is used to find the basics of compound Nd_2_Zr_2_O_7_ band structure in both the space groups for the spin up and spin down, for which we had to focus on the contributions of orbitals of all the atoms to the electronic density of states. 

Calculated values for total DOS show a number of noticeable features (peaks and valleys). In reality, we can detect the flawless fit and correlation between these peaks and the band structure. The calculated electronic band structure at the equilibrium lattice was constant for different high-symmetry points in the Brillouin zone and the total density of states DOS of Nd_2_Zr_2_O_7_ in GGA+U, respectively, where the line at zero eV indicates the Fermi energy.

**Figure 4:**
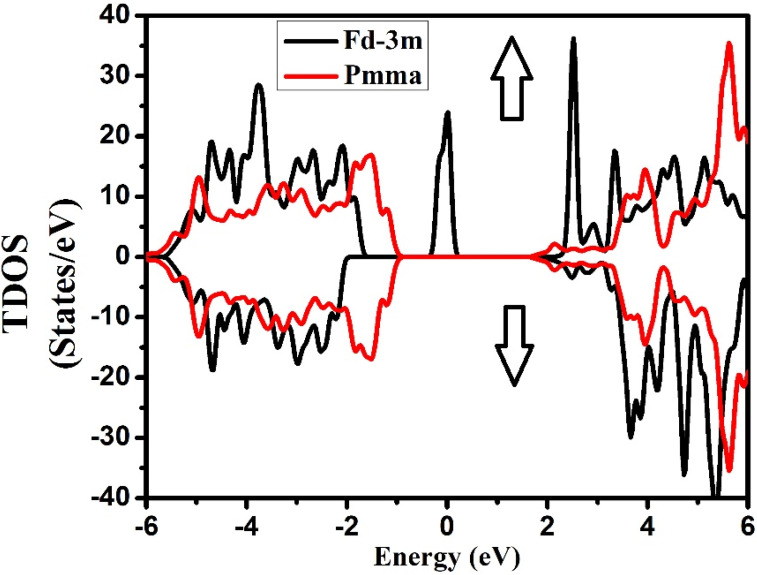
Total density of states for Nd_2_Zr_2_O_7_ in Fd-3m and Pmma phases for the spin-up direction (↑) and spin-down (↓) direction.

**Figure 5:**
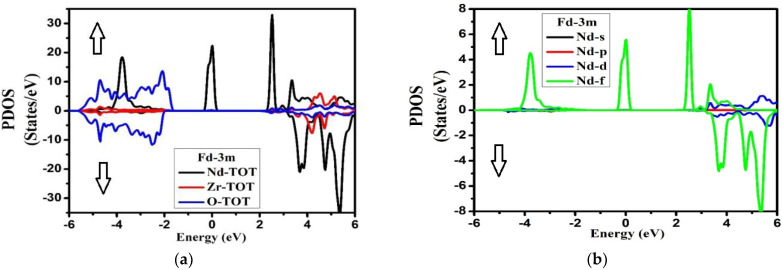

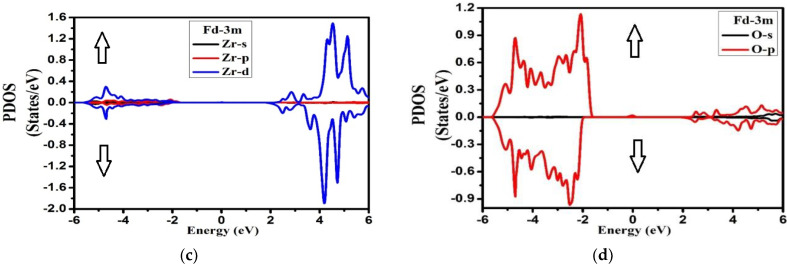
Partial density of states (**a**) Nd-TOT, Zr-TOT, O-TOT; (**b**) Nd-s,p,d,f; (**c**) Zr-s,p,d; (**d**) O-s,p for Nd_2_Zr_2_O_7_ in the Fd-3m phase for the spin-up direction (↑) and spin-down (↓) direction.

**Figure 6:**
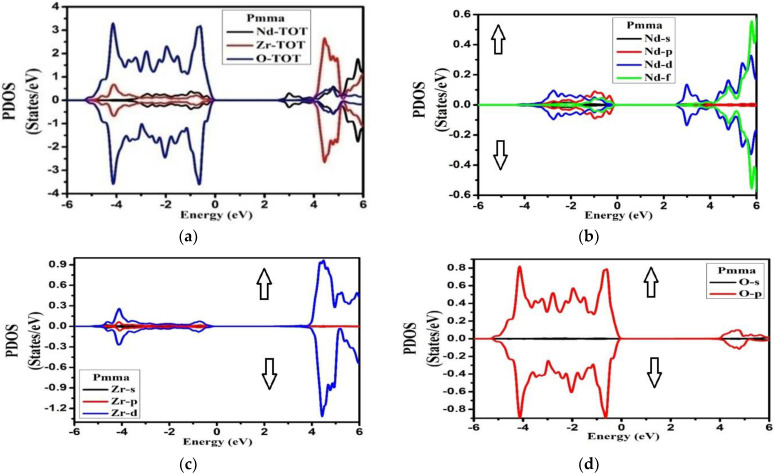
Partial density of states (**a**) Nd-TOT, Zr-TOT, O-TOT; (**b**) Nd-s,p,d,f; (**c**) Zr-s,p,d; (**d**) O-s,p for Nd_2_Zr_2_O_7_ in the Pmma phase for the spin-up direction (↑) and spin-down (↓) direction.

The TDOS (Figure 4) for both symmetries of the compound exhibited well-known energy regions in the valence band (VB) from −5.0 eV to −2.0 eV for the Fd-3m phase, while for Pmma it extended from −6.0 eV to −1.0 eV for both the spins, with a well-defined peak observed at 0.0 eV for Fd-3m phase. The VB was formed by the hybridization of the three atoms in the compounds, whose contributions can be found from the plots of PDOS in Figure 5 and Figure 6. The conduction bands (CB) extending from 2.0 eV to 6.0 eV for both the phases and both the spins were formed due to the hybridization of the three species.

From the plot of PDOS in Figure 5, we can see that, for the Fd-3m phase, the two peaks at −4.0 to −3.0 eV and at Fermi level originated from the Nd-f electronic states for the spin-up case. A large part of VB from −5.0 eV to −2.0 eV appeared due to O-p states. Meanwhile in CB, a prominent peak appeared at 2.5 eV from Nd-f states in the spin-up case too. From energy region 3.0 eV to 6.0 eV, the main contributions in the conduction band also arose from Nd-f states in the spin-down case with some considerable contribution from Zr-d states for both up and down spin cases. However, for the Pmma phase, the main contribution in VB was due to O-p states extending from −5.0 eV to −0.5 eV, as shown in Figure 6. The CB was mainly formed from Nd-(d + f) and Zr-d states extending from 4.0 eV to 6.0 eV with equal weightage from both spin states. The hybridization showed the strong covalent bonding between the species. This aspect suggests that there was a very strong electronic interaction between Nd-f, Nd-d, and Zr-d atomic orbitals. The considerable role of Nd-f and Zr-d beneath the Fermi level showed that it can donate the electrons. The contribution of the lower group of the conduction band was mostly due to Nd-f, Zr-d, and Zr-p orbitals, while in the Pmma phase, the peaks were generally due to Nd-d, Zr-d, and Zr-p electronic orbitals. The most important thing is that we have investigated the arrangement of the orbitals for all the nonequivalent locations for each element in the Fd-3m and Pmma phases of the Nd_2_Zr_2_O_7_ compound and important differences between the two phases have been noted. From this investigation, it has been ascertained that the peaks in the Fd-3m phase were higher than the Pmma phase and the structure moved to higher energies as we shifted from the Fd-3m phase to the Pmma phase. For Fd-3m phase, the valance band was mostly due to Nd-f states, while on the other hand, for Pmma, phase, it was due to Nd-d and Zr-d electronic orbitals.

### 3.4. Optical Properties

The optical behaviors of materials are generally derived from the band of structure electrons. The investigated optical dispersion properties provide important complementary information about the light-matter interaction behavior of the materials. These properties give us information about the filled and unfilled electronic states within the band [20]. The optical behavior plays a significant role in the potential opto-electronic appliances. From the real, ε_1_(ω), and the imaginary, ε_2_(ω), parts of complex dielectric function, we derive the essential optical properties, such as the extinction coefficient *K*(ω), refractive index *n*(ω), reflectivity (ω), absorption coefficient *I*(ω), energy-loss function *L*(ω), and real optical conductivity σ(ω) for both the phases. Here, we investigated the optical properties of the Nd_2_Zr_2_O_7_ compound for both the phases (Fd-3m and Pmma) at both the spins.

**Figure 7:**
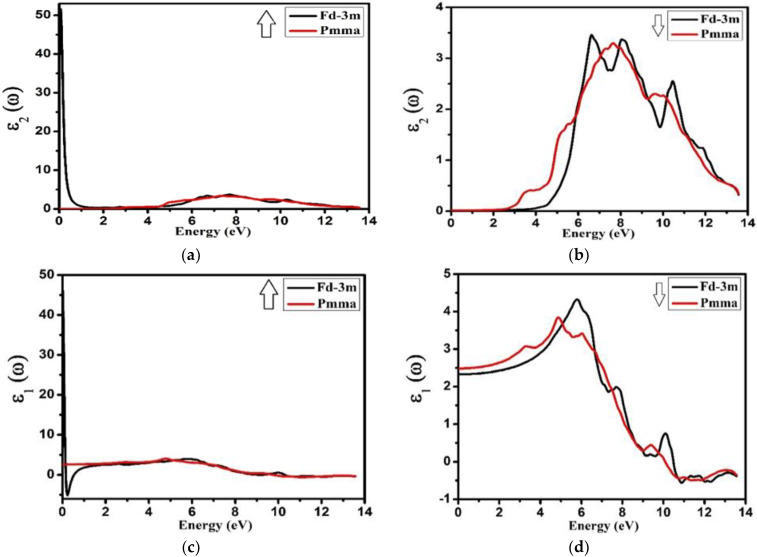
(**a**,**b**) The imaginary part, ε_2_(ω), of the complex dielectric function for Nd_2_Zr_2_O_7_ and (**c**,**d**) the real part, ε_1_(ω), of the complex dielectric functions for Nd_2_Zr_2_O_7_ ((black color for Fd-3m) and (red color for Pmma)) using GGA+U (**a**,**c**) for the spin-up direction (↑) and (**b**,**d**) spin-down (↓) direction.

Figure 7a is a plot of the investigated imaginary ε_2_(ω) part of the complex dielectric functions for both the phases at spin up and spin down, respectively. For both phases and both the spins, the real part of the dielectric function, ε_1_(ω), is shown in Figure 7b. The imaginary part, ε_2_(ω), of the complex dielectric function (DF) is related directly with the energy band structure. For the electron–phonon interaction, the broadening was considered to be 0.1 eV (used commonly). The peaks points for the spin up were approximately located at 7.9 eV for both the phases (Fd-3m and Pmma), while on the other hand, for spin-down, these points were positioned at 6.0 and 7.2 eV for Fd-3m and Pmma symmetries, respectively. The imaginary part of the complex dielectric function ε_2_(ω) also correlated with the transition of electrons between the bands of the similar momenta and its intensity was related with the overlapping between initial and final states. As permitted by the selection rules, the optical transition of electrons was allowed only between s to p, p to d, and d to p orbitals. A band between the energy values 5.0 to 9.0 eV corresponded to the electronic transitions near the highly symmetric point (Ӷv−Ӷc) in between the maximum of valence and the minimum of conduction bands, i.e., from the Op orbital in the valence band to the Zrd orbital in the conduction band for both the Fd-3m and Pmma phases. 

It can be seen that the spectra of the dielectric function of all materials studied were practically similar with a few simple differences. The reason for these differences was the discrepancies in the dispersion of the energy bands of these compounds. There was a sharp increase in the imaginary part, ε_2_(ω), of the dielectric function in between 5.0 eV and 9.0 eV for both the phases at spin down. Our observations showed that, as a whole, spectral shapes of the imaginary part for both phases were almost the same but the peaks for spin down were narrow as compared to spin up. The main reason behind this was the difference of hybridization of angular momenta of the two different phases, which changed the electronic transition and gave the spectral shape. The real part of the dielectric constant was related with the crystal polarization and gave a linear response of the substance to electro-magnetic radiation. It was also directly related with inter-band electronic dipole transition probability. Under the limitation of existence selection, rules of electronic transitions, the DF was related to the joint DOS near the Fermi level and the related part of the electronic band structure. 

The peaks value of ε_1_(ω) for spin-up were 4.9 and 6.0 eV, and for spin down were 5.0 and 6.0 eV, respectively, for the Fd-3m and Pmma phases. Our investigations showed that the values for real part of complex dielectric function ε_1_(ω) showed an increasing tendency, up to 5.0 eV for both phases, and a further increase in energy sets and a decrease in ε_1_(ω) for spin down. On the other hand, for spin up, the ε_1_(ω) value remained constant up to 6.0 eV and showed a slight decrease after 6.0 eV. The zero-crossing of the spectrum of the dielectric function’s dispersive part, ε_1_(ω), signified the non-existence of light scattering. Note that the function ε1(ω) vanished at a very small energy value for spin up and at 8.5 eV for spin down. For these energy values, the dispersion of light was zero and the value of the electronic part equal to ε_1_(ω) was obtained by using the dielectric constant ε_1_(ω) (ω→0), which is a critical parameter in many aspects of the characteristic material.

From ε_1_(ω) and ε_2_(ω) dispersions, the other optical properties, like extinction coefficient *K*(ω), absorption coefficient *I*(ω), refractive index *n*(ω), optical reflectivity *R*(*u*), and the energy-loss spectra *L*(*u*), can be calculated. 

**Figure 8:**
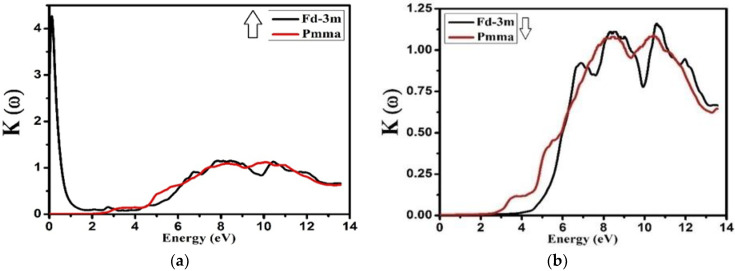
Calculated extinction coefficient *K*(ω) for Nd_2_Zr_2_O_7_ ((black color for Fd-3m) and (red color for Pmma)) using GGA+U. (**a**) The spin-up direction (↑) and (**b**) spin-down (↓) direction.

The extinction coefficient *K*(ω) determines how easily an electromagnetic radiation of a particular frequency (energy) can enter a material. The extinction coefficient *K*(ω) is related to the absorption of radiations, i.e., absorption coefficient *I*(ω). Figure 8 shows the extinction coefficient of (Fd-3m and Pmma) Nd_2_Zr_2_O_7_ for spin up and spin down, respectively. It is noted that the peak values of the extinction coefficient were obtained almost at 8.0 eV for both the phases and both the spins—for the spin up the band was broad but for a spin down the band was narrow. This is the indication that at this specific energy, the light was mostly absorbed by both the phases for spin up and spin down. After 11.0 eV, the value of extinction went on to decrease, which means that Nd_2_Zr_2_O_7_ was transparent for energy values greater than 11.0 eV.

**Figure 9:**
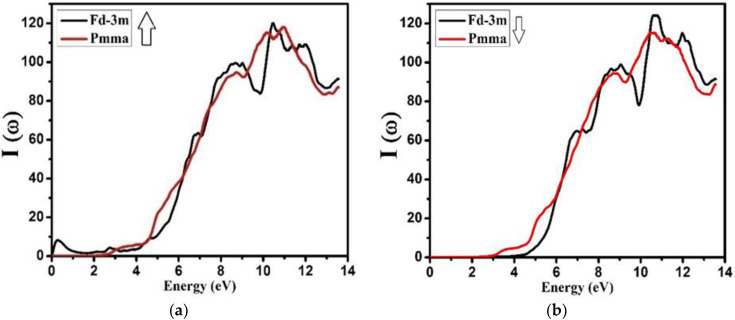
Calculated absorption coefficient *I*(ω) of Nd_2_Zr_2_O_7_ ((black color for Fd-3m) and (red color for Pmma)) using GGA+U. (**a**) The spin-up direction (↑) and (**b**) spin-down (↓) direction.

The absorption spectra are related to the absorption of electromagnetic radiations of definite wave lengths or frequencies by the compounds. Figure 9 illustrates the absorption spectra of NZO compound for both phases (Fd-3m and Pmma) at spin up and spin down. For both the Fd-3m and Pmma symmetries, no photons were absorbed for spin up (ћω < 2.0 eV in Fd-3m and ћω < 2.5 eV in Pmma) or for spin down (ћω < 2.5 eV, ћω < 4.5 eV) (see Figure 9), respectively. From the investigation, it was concluded that the highest absorption peaks and the broad absorption spectrum were observed above 9.0 eV for both spin up and down of Fd-3m and Pmma phases, which represents the formation of the electronic transition from the valence to the conduction band of the material. The absorption spectra started decreasing from 11.0 eV, which means that the material is not suitable for the ultraviolet region. From the investigation, it was also observed that the absorption spectra increased directly with small fluctuating peaks against energy from 2.9 to 10.8 eV for both the phases at spin up and for spin down (3.0 to 10.9 eV for Fd-3m and 4.5 to 11.0 eV for Pmma).

**Figure 10:**
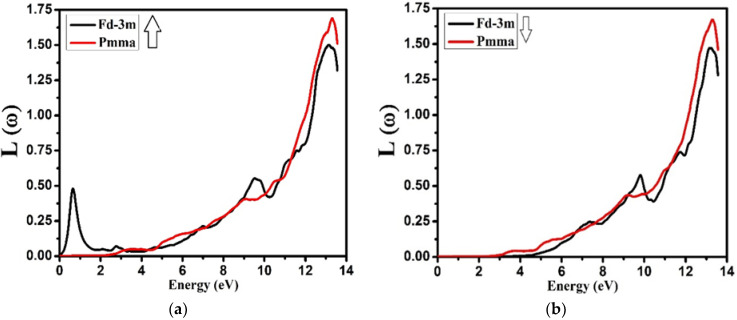
Calculated energy-loss function *L*(ω) for Nd_2_Zr_2_O_7_ ((black color for Fd-3m) and (red color for Pmma)) using GGA+U. (**a**) The spin-up direction (↑) and (**b**) spin-down (↓) direction.

Figure 10 represents the energy-loss function *L*(ω) of NZO compound for both the phases and both the spins. It is considered to be an imperative optical parameter and is used to measure the energy loss of very highly energetic electrons passing through the material. The peaks values of the energy loss function *L*(ω) signify the features associated with the plasmonic vibrational frequencies and are also said to be plasma oscillations/ frequencies.

From the investigation, it was observed that the peak values of *L*(ω) were in between 13.0 and 13.3 eV for the Fd-3m and Pmma phases at both spin up and down. The peaks that were dominant for the *L*(ω) spectra occurred due to the excitations of plasmon. The reason beyond is the combined longitudinal oscillatory response of the electrons in the valence band against the atomic cores with plasma frequency.

**Figure 11:**
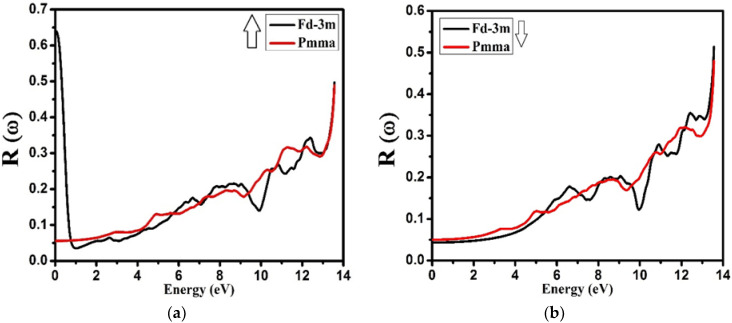
Calculated reflectivity *R*(ω) for Nd_2_Zr_2_O_7_ ((black color for Fd-3m) and (red color for Pmma)) using GGA+U. (**a**) The spin-up direction (↑) and (**b**) spin-down (↓) direction.

The reflectivity dispersion of the NZO compound for both the phases and both the spins is shown in Figure 11. For spin up, the value of reflectivity was 0.65 at energy 0.0 eV, while a rapid decrease up to 0.03 was shown in reflectivity for the energy values from 0.0 eV to 1.0 eV. The further increase in energy corresponded to a gradual increase in reflectivity with small fluctuating peaks up to 12 eV for the Pmma phase, with the reflectivity varying between 0.06 to 0.35 for the energy 0.0 eV to 12 eV. Similarly, for the spin down, the reflectivity for both the phases varied between 0.05 to 3.5 for the energy from 0.0 eV to 12.4 eV. Above this energy, the reflectivity for both the phases and both the spins showed a rapid increase. From the investigation, it was observed that for higher energies, the reflectivity was also high for both the phases of the compound. Thus, we can say that the results are coincident with energy loss spectrum, falling in the range of 4.0 eV to 12.0 eV. 

**Figure 12:**
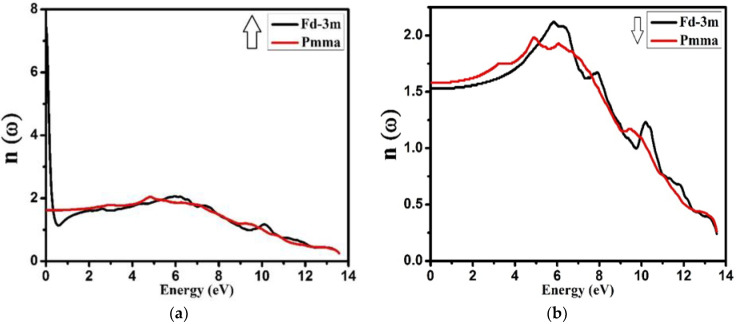
Calculated refractive index *n*(ω) for Nd_2_Zr_2_O_7_ ((black color for Fd-3m) and (red color for Pmma)) using GGA+U. (**a**) The spin-up direction (↑) and (**b**) spin-down (↓) direction.

The refractive index by the definition is “actually the ratio between the speed of light in free space and the speed of light in that material”. Figure 12 shows the refractive index variation of both the phases and both the spins. For both the phases, in both spin up and spin down, it was observed that below 10.0 eV and 6.0 eV, the index of refraction lay between 1 and 2. This indicates that the material is transparent to the visible spectrum of light. The maximum refractive indexes for both the spins and for both (Fd-3m and Pmma) phases were found at around 6.0 eV.

**Figure 13:**
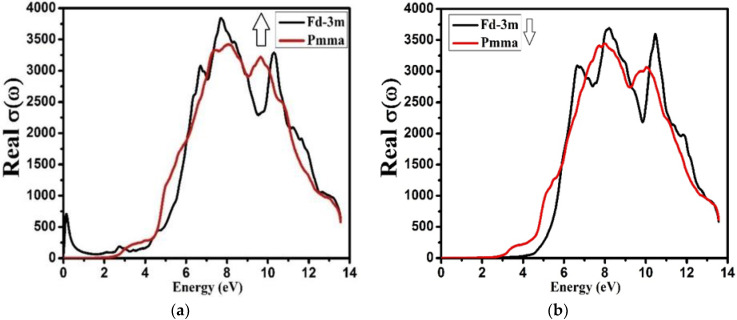
Calculated real optical conductivity *σ*(ω) for Nd_2_Zr_2_O_7_ ((black color for Fd-3m) and (red color for Pmma)) using GGA+U. (**a**) The spin-up direction (↑) and (**b**) spin-down (↓) direction.

The term real optical conductivity *σ*(ω) means the ability of a material to conduct electric charge under the influence of electro-magnetic (EM) radiations. The term “optical”, here, means the whole spectrum of the electromagnetic radiation, not constrained only to the visible region of EM radiations. The real optical conductivity *σ*(ω) values for both the phases, Fd-3m and Pmma, for spin up and spin down, are shown in Figure 13. It was observed that, for spin up, the real optical conductivity was zero for the energy less than 2.5 eV for both the phases, and started increasing by increasing the energy up to 7.0 eV.

With further increases in energy, the conductivity started decreasing, meaning that the material behaved like a conductor for the energy range between 2.5 to 11eV. On the other hand, for spin down, the optical conductivity was zero for less than 2.5 and 4.3 eV for the phases Fd-3m and Pmma, respectively. For the energy increasing up to 8.0 eV, the conductivity increased gradually, and started to decrease with further increase in energy. 

## 4. Conclusions

We have investigated the structural, electronic, and optical properties of Nd_2_Zr_2_O_7_ for both (spin up and spin down) Fd-3m and Pmma phases via DFT-based WIEN2k calculations. The compound Nd_2_Zr_2_O_7_ exhibits a direct band gap. The calculated band gaps for spin up were 2.09 eV and 2.39 eV, and for spin down were 3.90 eV and 2.40 eV, respectively, for Fd-3m and Pmma. From the electronic band structure, it is clear that the electronic density of states (DS) near to the Fermi level is mainly caused by the Nd-f and Zr-d states for the Fd-3m phase, while for Pmma phases the contribution is mainly due to Nd-d, Nd-f, and Zr-d, which is the confirmation of covalency of a certain level between Nd-f and Zr-d chemical bonding. This aspect suggests a very strong electronic interaction between Nd-f, Nd-d, and Zr-d atoms. The extinction coefficient reached its peak at almost 8.0 eV for spin up and spin down of both the phases; for the spin up, the band was broad, but for spin down, the band was narrow. The dominant peaks observed for the energy-loss function spectra were due to plasma excitation of electrons. For both the phases and both the spins, the compound Nd_2_Zr_2_O_7_ possessed a comparatively high value of reflectivity and absorption in the spectrum of the UV region. The refractive index has been studied within the energy range 0.0 eV to 14.0 eV. In real optical conductivity for both the phases, Fd-3m and Pmma (spin up and spin down), it was observed that for the spin up the real optical conductivity was zero for energy levels less than 2.5 eV for both the phases and started increasing by increasing the energy up to 7.0 eV, after which, further increases in energy led the conductivity to start decreasing. On the other hand, for spin down, the optical conductivity was zero for less than 2.5 and 4. Fd-3m and Pmma, respectively, and as the energy increased up to 8.0 eV, the conductivity increased gradually, after which it started to decrease with further increases in energy. This means that the material behaves like a conductor for the energy range between 2.5 eV to 11.0 eV. In conclusion, we are expecting that this theoretical study will motivate experts to explore the attractive properties of this oxide material in more detail in future, theoretically as well as experimentally.

## Figures and Tables

**Table 1:** Space group Fd-3m (227), lattice parameters, and fractional coordinates.

Atoms	X	Y	Z
Nd	0.12500000	0.12500000	0.12500000
Nd	0.62500000	0.12500000	0.12500000
Nd	0.12500000	0.12500000	0.62500000
Nd	0.12500000	0.62500000	0.12500000
Zr	0.62500000	0.62500000	0.62500000
Zr	0.12500000	0.62500000	0.62500000
Zr	0.62500000	0.62500000	0.12500000
Zr	0.62500000	0.12500000	0.62500000
O	0.00000000	0.00000000	0.00000000
O	0.25000000	0.25000000	0.25000000
O	0.28956700	0.28956700	0.71043300
O	0.53956700	0.53956700	0.96043300
O	0.71043300	0.28956700	0.71043300
O	0.71043300	0.28956700	0.28956700
O	0.28956700	0.71043300	0.71043300
O	0.28956700	0.71043300	0.28956700
O	0.71043300	0.71043300	0.28956700
O	0.96043300	0.53956700	0.53956700
O	0.53956700	0.96043300	0.96043300
O	0.96043300	0.96043300	0.53956700
O	0.96043300	0.53956700	0.96043300
O	0.53956700	0.96043300	0.53956700

data_Nd_2_Zr_2_O_7_; space group Fd-3m (227); a: 7.63484829; b: 7.63484829; c: 7.63484829; α = 60.00; β = 60.00; γ = 60.00.

**Table 2:** Space group Pmma, lattice parameters, and fractional coordinates.

Atoms	X	Y	Z
Nd	0.50000000	0.50000000	0.50000000
Nd	0.00000000	0.75000000	0.22231000
Nd	0.50000000	0.00000000	0.50000000
Nd	0.00000000	0.25000000	0.77769000
Zr	0.00000000	0.25000000	0.23880500
Zr	0.00000000	0.75000000	0.76119500
Zr	0.50000000	0.00000000	0.00000000
Zr	0.50000000	0.50000000	0.00000000
O	0.00000000	0.46145300	0.63341100
O	0.50000000	0.75000000	0.86688600
O	0.00000000	0.52504300	0.88566600
O	0.00000000	0.47495700	0.11433400
O	0.00000000	0.02504300	0.11433400
O	0.50000000	0.75000000	0.10071700
O	0.50000000	0.25000000	0.36987600
O	0.00000000	0.53854700	0.36658900
O	0.00000000	0.96145300	0.36658900
O	0.00000000	0.03854700	0.63341100
O	0.50000000	0.75000000	0.63012400
O	0.50000000	0.25000000	0.89928300
O	0.00000000	0.97495700	0.88566600
O	0.50000000	0.25000000	0.13311400

data_Nd_2_Zr_2_O; space_group Pmma; a: 3.67429700; b: 7.67588500; c: 10.99859600; α = 90.00000000; β = 90.00000000; γ = 90.00000000.

## Data Availability

Not applicable.
